# Geographic Differentials in Mortality of Children in Mozambique: Their Implications for Achievement of Millennium Development Goal 4

**DOI:** 10.3329/jhpn.v30i3.12297

**Published:** 2012-09

**Authors:** Gloria Macassa, Gebrenegus Ghilagaber, Harry Charsmar, Anders Walander, Örjan Sundin, Joaquim Soares

**Affiliations:** ^1^Department of Occupational and Public Health Sciences, University of Gävle, 80176 Gävle, Sweden; ^2^Department of Public Health Sciences, Division of Social Medicine, Karolinska Institute, SE-17176, Sweden; ^3^Division of Public Health Sciences, Mid-Sweden University, 85170, Sundsvall, Sweden; ^4^Department of Statistics, Stockholm University, SE-106 91, Sweden; ^5^Department of Sociology, Stockholm University, SE-106 91, Sweden; ^6^Department of Psychology, Mid-Sweden University, 83125 Östersund, Sweden

**Keywords:** Child mortality, Economic development, Infant mortality, Millennium Development Goals, Mozambique

## Abstract

In the light of Mozambique's progress towards the achievement of Millennium Development Goal 4 of reducing mortality of children aged less than five years (under-five mortality) by two-thirds within 2015, this study investigated the relationship between the province of mother's residence and under-five mortality in Mozambique, using data from the 2003 Mozambican Demographic and Health Survey. The analyses included 10,326 children born within 10 years before the survey. Results of univariate and multivariate analyses showed a significant association between under-five mortality and province (region) of mother's residence. Children of mothers living in the North provinces (Niassa, Cabo Delgado, and Nampula) and the Central provinces (Zambezia, Sofala, Manica, and Tete) had higher risks of mortality than children whose mothers lived in the South provinces, especially Maputo province and Maputo city. However, controlling for the demographic, socioeconomic and environmental variables, the significance found between the place of mother's residence and under-five mortality reduced slightly. This suggests that other variables (income distribution and trade, density of population, distribution of the basic infrastructure, including healthcare services, climatic and ecologic factors), which were not included in the study, may have confounding effects. This study supports the thought that interventions aimed at reducing under-five mortality should be tailored to take into account the subnational/regional variation in economic development. However, research is warranted to further investigate the potential determinants behind the observed differences in under-five mortality.

## INTRODUCTION

In September 2000, Mozambique joined other countries in signing the declaration which launched the United Nations Millennium Development Goals (MDGs), with 1990 scenario as baseline. Of the eight MDGs, Goal 4 aims at reducing mortality of children aged less than five years (under-five mortality) by two-thirds within 2015. However, as stated by the UN report on the progress of the MDGs in 2010, child deaths are falling but not quickly enough to reach the target (1). The report found that, overall, sub-Saharan Africa still experiences high levels of under-five mortality compared to other regions of the world, although a reduction has occurred from 184 per 1,000 livebirths in 1990 to 144 per 1,000 livebirths in 2008 (1). Further, the report found that some countries in the sub-Saharan Africa region have achieved absolute reductions in under-five mortality against the odds of poverty. These countries include Mozambique, Ethiopia, and Malawi (1).

Although the baseline figures for mortality in 1990 are not available for Mozambique, the most recent country report on the progress towards the achievements of MDGs shows that under-five mortality has declined from 219 per 1,000 livebirths in 1997 to 178 per 1,000 in 2003 (1,2). In addition, data from the most recent Multiple Indicator Cluster Survey (MICS) showed that a further reduction has taken place, and in 2008, under-five mortality reached 154 per 1,000 livebirths (3). However, despite this positive development in under-five mortality rates at the national level, there is a growing concern regarding the persisting geographical differences in under-five mortality (1) (Table 1).

The 2010 country report on the MDGs for Mozambique noted that there were great geographical differences in under-five mortality. A child in the North province of Cabo Delgado was three times more likely to die before the age of five years than a child born in Maputo city (1). According to the report, infant mortality was lower in the Southern region compared to the Central and Northern regions of the country, with the mortality rate of 147 per 1,000 livebirths in Zambezia and 131 per 1,000 livebirths in Cabo Delgado (1). The rates of child mortality were 180 per 1,000 livebirths and 205 per 1,000 livebirths in Cabo Delgado and Zambezia respectively (1). Maputo city had a child mortality rate of 108 per 1,000 livebirths, and Maputo province had a child mortality rate of 103 per 1,000 livebirths. The report noted that the country had a potential to achieve its overall 2015 targets for child mortality (67 per 1,000 livebirths) and under-five mortality (108 per 1,000 livebirths) (1). However, no breakdown for the child mortality and under-five mortality targets by province was given.

**Table. 1.** Under-five mortality rates per 1,000 livebirths by province

Province/region	1997 Mozambique Demographic and Health Survey	2003 Mozambique Demographic and Health Survey	Multiple Cluster Survey 2009
Niassa	213	206	123
Cabo Delgado	165	241	180
Nampula	319	220	140
Zambezia	183	123	205
Tete	283	206	174
Manica	159	184	154
Sofala	242	205	130
Inhambane	193	149	117
Gaza	208	156	165
Maputo	147	108	103
Maputo city	97	89	108
National under-five mortality rate	219	179	154

Source: Mozambique National Institute of Statistics, 2010

Geographically, the country exhibits substantial differences in welfare and economic development, with a high concentration of economic activities, infrastructure, and basic services (including healthcare facilities) in and around the capital Maputo city, situated in the very south of the country (4) (Table 2). This has resulted in differences in the development of regional welfare, which are important issues in Mozambican society and politics.

To understand the role of province (region) of residence in the differentials of under-five mortality in Mozambique, this study relies on the framework of the proximate determinants by Mosley and Chen (5). The model of the proximate determinants was developed to study the factors affecting child mortality and is based on the idea that all social and economic determinants of child mortality operate through a set of biological or proximate determinants to affect a child's probability of survival (5). The model combined social, economic, medical and biological explanations of child mortality. Mosley and Chen (5) grouped the proximate determinants into five categories: (i) maternal factors (age, parity, and birth interval); (ii) environmental contamination (air, food/water/fingers, skin/soil/inanimate objects, and insect vectors); (iii) nutrient deficiency (calories, proteins, and micronutrients, such as vitamins and minerals; (iv) injury (accidental or intentional); and (v) personal illness control (personal preventive measures and medical treatment). All the social and economic determinants of child mortality—the 'distal’ determinants—operate through these proximate determinants and are grouped by Mosley and Chen into individual-level, household-level and community-level variables (5).

**Table. 2.** Distribution of selected social, economic and health indicators by province/region

Indicator	National	South region				Central region				North region		
		Maputo city	Maputo province	Gaza	Inhambane	Sofala	Manica	Tete	Zambezia	Nampula	Cabo Delgado	Niassa
Population–NIS projection for 2004	19 million	1,073,940	1,074,790	1,333,540	1,140,220	1,582,260	1,280,830	1,461,650	3,645,630	3,563,220	1,588,740	966,580
Children aged below 18 years (2004)	9,613,470	473,550	496,080	647,320	690,120	790,270	675,940	799,495	1,911,980	1,832,340	777,070	519,330
% of population living below poverty-line (2003)	54	53.6	69.3	60.1	80.7	36.1	44.6	59.8	44.6	52.6	63.2	52.1
Mortality												
Under-five mortality rate (2003)	178	89	108	156	149	205	184	206	123	220	241	206
IMR (2003)	124	51	61	92	91	149	128	125	89	164	178	140
Nutritional status (%)												
Chronic malnutrition among children aged 0-5 year(s) (stunting) (2003)	41	21	24	34	33	42	39	46	47	42	56	47
Acute malnutrition among children aged 0-5 year(s) (wasting) (2003)	4	0.8	0.5	6.7	1.3	7.6	2.8	1.6	5.2	6.0	4.1	1.3
Underweight children aged 0-5 year(s) (2003)	23.7	7.9	9.2	22.6	12.8	26.2	22.9	25.1	26.9	28.2	34.2	25.1
Water and sanitation (%)												
Access to safe drinking-water (2003)	35.7	66.2	48.9	50.2	31.6	47.7	47.1	41.6	13.7	32.2	41.6	30.2
Access to sanitation (2003)	44.8	99.7	90.2	69.4	66	28.8	45.6	42.7	19.2	26.2	53.1	70
HIV/AIDS prevalence among persons aged 15-49 years (2002)	13.6	17.3	17.4	16.4	8.6	26.5	19	14.2	12.5	8.1	7.5	11.1
Immunization (%)												
Children, aged 12-23 months, fully immunized (DPTHepB) (2003)	63.3	91.3	92.5	82.3	90.6	63.9	61.6	55	44.7	53.9	57.9	46.6
Children, aged 12-23 months, immunized against measles (2003)	76.7	96.9	96.9	91.7	92.9	74.7	81.5	72	63.3	69.1	80.2	51.9
Education and illiteracy (%)												
Primary net enrollment rate (2003)	61	84.5	86	79.2	74	60.4	67	52.1	59.8	46.3	60.6	47.3
Adult illiteracy rate (2003)	53.6	15.1	28.6	47.4	46.5	52.7	45.4	59.2	61.4	65.1	68.4	64.4
Female illiteracy rate (2003)	68	22	38	55.9	57.9	72.2	64.5	76.1	80.6	81.4	83	68
Maternity care and adolescent fertility (%)												
Fertility rate (2003)	5.5	3.2	4.1	5.4	4.9	6	6.6	6.9	5.3	6.2	5.9	7.2
Births attended by skilled health personnel (2003)	47.7	89.2	85.2	60.6	49	51	55.9	46.8	32.1	38.2	31.4	47
Births in health institutions (2003)	49	90.1	85.4	63.1	49.8	51.6	56.0	47.4	32.7	36.8	29.6	46
Communication												
Total % of population with radios (2003)	45.5	61.8	53.4	34.1	32.9	52.3	63.6	45.1	39.4	48.3	43	43

Source: World Bank, 2010.

IMR=Infant mortality rate;

NIS=National Institute of Statistics

Although there is no general agreement on the theory for the relationship between the region of residence and under-five mortality (6,7), we hypothesize that the province of mother's residence is a potential community variable that may reflect the environmental, social and socioeconomic factors, which influence under-five mortality. In addition, we see the province of mother's residence as proxy for inequalities in the underlying social, economical, cultural, ethnic and climatic factors but inequalities in provision of health services (including availability and access to health services and medical assistance) are seen crucially. Thus, the province of mother's residence would impact under-five mortality in Mozambique through maternal factors, such as age, parity, and birth interval; environmental contamination (water supply); health behaviours and practices; and use of healthcare services.

To the best of our knowledge, no study has attempted to investigate the differentials in under-five mortality by province in Mozambique to date, although it is known that the country has experienced a very unequal geographical socioeconomic development. Therefore, as Mozambique progresses towards 2015, this study aims to assess the role of mother's province/region of residence on the risk of under-five mortality.

## MATERIALS AND METHODS

### Study site

Mozambique, located on the east coast of southern Africa bordering Tanzania in the North, South Africa and Swaziland in the South, and Zimbabwe, Zambia, and Malawi in the West, is one of the poorest countries in sub-Saharan Africa (Development Index of 165 among 182 countries in 2010) and in the world (2).

The number of inhabitants has increased from 16 million in 1997 to about 21 million in 2010 (2). The provinces of Zambezia (Central region) and Nampula (North region) are most populated, with 2,891,809 and 2,975,747 inhabitants respectively whereas Niassa (North region) has a population of 756,287 (2).

Although Mozambique, since the end of the civil war in 1992, has had an annual economic growth rate of 8% on average (4), the country is still very poor with a gross domestic product (GDP) of about PPP$ 900 per capita (1), and around 54% of the population lives under the poverty-line (4). Life-expectancy at birth for both the sexes is 52.9 years (UNDP factsheet 2011), and the literacy rate is 54% (69.5% for males and 40.1% for females). In 2008, the net rates of primary school enrollment were 92% for male and 86% for female (2).

Mozambique is predominantly a rural country, with 69% of the population living in rural areas compared to 31% in urban areas. The country depends on subsistence farming, and the agricultural sector represents 20% of the total GDP of the country (4).

The population of Mozambique consists mainly of indigenous tribal groups, such as Shangaan, Chokwe, Manyika, Sena, Makwa, and others. Europeans comprise only 0.06% of the population, Euro-Africans 0.2%, and Indians 0.08% (4,8,9). The official language is Portuguese but only 24.3% can speak and write in it. The majority of the population speaks one of the 13 national languages, with Makwa (27.8%) and Tsonga (12.4%) being the most common spoken languages. Islam is the predominant religion in the Northern provinces and in the central coastal area while the Catholic and the Protestant religions prevail in the South and in the interior of the Central provinces. However, many Mozambicans are still Animist, believing in Alma and Spirits (4,8). The country is divided into three regions: North region (Cabo Delgado, Niassa, and Nampula), Central region (Sofala, Zambezia, Manica, and Tete), and South region (Inhambane, Gaza, Maputo, and Maputo city) (9) (Fig.).

Although Mozambique achieved a considerable economic growth in the 1990s, the benefits of economic development have not been distributed evenly (4,8). Poverty remains endemic, and sharp inequalities exist in the country. For instance, in 1997, the national Gini coefficient was 40, which increased to 42 in 2002 (4).

### Study sample

Data used in the study were drawn from the 2003 Mozambique Demographic and Health Survey (MDHS) conducted during August–December 2003. The sample included 12,418 women aged 15-49 years. Information was collected on birth-history, personal and household characteristics, health service-use, and child health at the time of the survey. From the individual mother's file, a retrospective child's file consisting of all children born to sample women was generated. Each livebirth and the subsequent health outcome contain information on the household and parents. With this transformation, child's records constituted the basic analytic sample. Since women may have had multiple births before being interviewed, the child's file includes siblings.

For the purpose of the study, analyses were restricted to children born within 10 years before the survey (n=10,326). The restriction served two purposes: (i) reduced recall error because the quality of the birth-history information for recent births was better than for more distant births and (ii) the assumption of a static risk profile using retrospective data was least violated when using recent births. The survey had a response rate of 82%. More details regarding the survey and its sampling were reported in the official document of the 2003 Mozambique Demographic and Health Survey (9).

**Fig.**
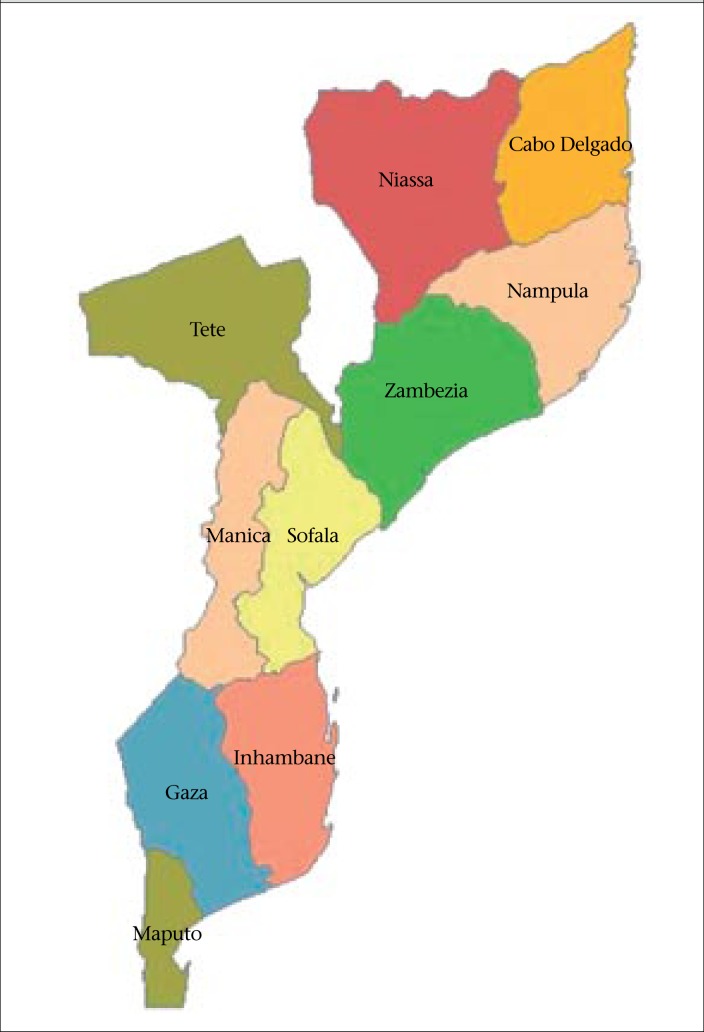
Map of Mozambique

### Specification and measurement of independent variables

#### Demographic variables

Sex of the child was classified as male or female. Age of the mother at the child's birth was grouped in 15-18, 19-23, 24-28, 29-33, and 34+ years. Birth order and the preceding birth interval were merged into one variable (birth order/birth interval) to enable the inclusion of first births in the analyses and were classified into seven categories: (i) first births; (ii) birth order 2-4 and short birth interval (<24 months), (iii) birth order 2-4 and medium birth interval (24-47 months), (iv) birth order 2-4 and long birth interval (48+ months), (v) birth order 5+ and short birth interval (<24 months), (vi) birth order 5+ and medium birth interval (24-47 months), and (vii) birth order 5+ and long birth interval (48+ months).

#### Social and economic variables

The province of the mother's residence was grouped as follows: North region (Cabo Delgado, Niassa, and Nampula), Central region (Sofala, Zambezia, Manica, and Tete), and South region (Inhambane, Gaza, Maputo, and Maputo city).

Religious affiliation of the mother was grouped as Catholic, Muslim, Zionist, Protestant/Evangelic, and Animist/others. The place of residence was classified into urban and rural, using *de jure* place of residence (where the mother legally resides).

**Parental education and occupation:** In the MDHS data (9), the parental education variable referred to the highest level of education attained. There were six categories, i.e. no education, primary incomplete, primary complete, secondary incomplete, secondary complete, and higher education. For the purpose of the study, three categories of the same variable were created: (i) no education, (ii) primary education, and (iii) secondary and higher education. There were 10 categories of parental occupation in the MDHS, i.e. not working, professional, technical, clerical, sales, agricultural (self-employed), agricultural (employed), service, skilled manual work, and unskilled manual work. For the purpose of the study, the occupational categories were merged into four groups: (i) professional; (ii) clerical, sales, service, and skilled manual worker; (iii) agricultural (self-employed), agricultural (employed), and unskilled manual worker; and (iv) not working.

**Wealth index:** The 2003 MDHS also included a household wealth status index which was estimated from several household characteristics and asset variables, using principal component analysis. The household characteristics used in estimating the household wealth index included having electricity, type of source of drinking-water, access to a sanitation facility, availability of cooking-fuel, main roof-material, main wall-material, and floor-material. The asset variables included durable goods (wardrobe, table, chair or bench, watch or clock, radio, television, bicycle, motorcycle, sewing machine, and telephone) and ownership of land. This household wealth index was used as a proxy indicator for household wealth status in the analyses. Household wealth inequality was measured by dividing the wealth index into quintiles, with the lowest quintile representing the poorest 20% of households and the highest quintile representing the wealthiest 20% of households in Mozambique. More details regarding the wealth index in the DHS can be found elsewhere (9,10). In this study, there are five quintiles: (i) the poorest; (ii) poorer; (iii) middle; (iv) richer; and (v) the richest.

#### Household and environmental variables

Three household and environmental variables, i.e. toilet facility, source of drinking-water, and type of floor material, were used in the study. Three categories were created for toilet facility: (i) flush toilet (own flush toilet, shared flush toilet); (ii) traditional toilet (traditional pit-toilet and latrine); and (iii) no toilet facility. For source of drinking-water, four categories were created: (i) piped water (piped into own residence, piped into neighbour's residence); (ii) public tap; (iii) well (well in residence, well in the neighbour's residence, public well); and (iv) others (spring, river, lake, dam, rainwater). Type of floor-material was categorized as: (a) natural (clay), (b) rudimentary (wood and adobe), and (d) finished. The indicators used in the study are described in Table 3.

### Methods

The dependent variable (outcome) was the risk of death of under-five children (0-59 months), and the main independent variable (exposure) was the province of mother's residence. Age of mother, birth order, birth interval, age of child, place of residence, education of mother, occupation of mother, religious affiliation of mother, source of drinking-water, type of floor-material, and type of toilet facility were used as control variables.

To estimate the effects of mother's residence on the risk of mortality for under-five children, we used Cox regression analysis (11) in the SPSS software (version 17) (12), and results were expressed as mortality risk ratios with 95% confidence intervals (Table 4). Respondents with missing values were excluded from the analysis. There were very few missing values (Table 3).

The analyses were performed using three models. Model I dealt with the univariate relationship between under-five mortality and province of mother's residence without adjusting for the influence of any other variables. In Model II, we adjusted for the demographic variables, such as age of mother, birth order, birth interval, and sex of child. In Model III, we further adjusted for the social and economic variables (urban-rural place of residence, parental education and occupation, wealth index, and religious affiliation of mother) and the environmental variables (source of drinking-water, type of toilet facility, and type of floor-material).

## RESULTS

Table 3 shows that children in the North (Niassa and Cabo Delgado) and Central (Zambezia, Manica, and Sofala) provinces had higher risks of mortality than those in the South, particularly Maputo city.

In the univariate model (with no adjustment for any other variable) (Model I, Table 4), compared to children in Maputo province, those in the North provinces of Cabo Delgado, Niassa, and Nampula had a risk of dying which was 2.89, 2.11, and 2.35 respectively. In addition, compared to children in Maputo city, those in the Central province of Zambezia had a risk of dying which was 2.43 times higher in Tete, 2.35 times higher in Sofala, and 1.81 times higher in Manica (Model I, Table 4). In the southern provinces, children in Inhambane had an under-five mortality risk which was 1.79 times higher than that of children in Maputo city (Model I, Table 4).

Controlling for the demographic variables (Model II, Table 4), the socioeconomic and household environment variables (Model III, Table 4) slightly reduced the risks obtained in the univariate analyses but, overall, the mortality risks continued to be significant. For instance, in Model II, the risk of under-five mortality for children in the northern province of Niassa decreased from 2.11 to 1.94 in Model III. Furthermore, the under-five mortality risk for children in the North province of Cabo Delgado decreased from 2.89 to 2.80 in Model III (Table 4). This pattern was also observed in the mortality risks of children in the Central and South provinces (Model II and III, Table 4). In the Central region, the mortality risk in Manica province decreased from 1.81 in Model I to 1.68 in Model III and in Sofala, from 2.35 in Model I to 2.12 in Model III (Table 4). In the South, the under-five mortality risks decreased only slightly in Gaza province, from 1.79 in Model I to 1.72 in Model III (Table 4).

## DISCUSSION

The results of the univariate and multivariate analyses showed a significant association between under-five mortality and the region of mother's residence. Children of mothers living in the North provinces (Niassa, Cabo Delgado, and Nampula) and in the Central provinces (Zambezia, Sofala, Manica, and Tete) had higher mortality risks than children whose mothers lived in the South region, especially in Maputo province and Maputo city. However, within the South provinces, children whose mothers resided in Inhambane province had slightly higher under-five mortality risks than children whose mothers were from Gaza and Maputo provinces.

Similar patterns of regional differences in under-five mortality have been reported in other developing countries, especially in sub-Saharan Africa (13-20). For instance, a study by Root in Zimbabwe reported that child mortality was 45% lower in Ndebele province than Shona province (18). Furthermore, a study by Uwazurike found sizeable district-specific geographic variations in the level of under-five mortality in Malawi, Nigeria, Tanzania, and Zambia (21).

**Table. 3.** Description of variables used in Cox regression analysis of under-five mortality by province of mother's residence, 2003 Mozambique Demographic and Health Survey

Variable	No.	%
Province of mother's residence (region)		
North region		
Niassa	837	8
Cabo Delgado	807	8
Nampula	1,174	11
Central region		
Zambezia	952	9
Tete	1,152	11
Manica	1,042	10
Sofala	1,138	11
South region		
Inhambane	846	8
Gaza	980	10
Maputo province	715	7
Maputo city	683	7
Total	10,326	100
Demographic variable		
Mother's age (years) at childbirth		
24-28	2,752	26.7
18 and less	603	5.8
19-23	2,686	26.0
29-33	1,993	19.3
34 and above	2,292	22.2
Total	10,326	100
Birth order—preceding birth interval months		
Order 2-4 and medium interval (24-47 months)	2,978	28.8
First births	2,353	22.8
Order 2-4 and shorter interval (<24 months)	782	7.6
Order 2-4 and long interval (48+ months)	1,080	10.5
Order 5+ and shorter interval (<24 moths)	469	4.5
Order 5+ and medium interval (24-47 months)	1,876	18.2
Order 5+ and long interval (48+ months)	770	7.5
Missing	18	0.1
Total	10,326	100
Sex of child		
Male	5,139	49.8
Female	5,187	50.2
Total	10,326	100
Socioeconomic variable		
Place of mother's residence		
Urban	3,639	35.2
Rural	6,687	64.8
Total	10,326	100
Wealth index		
Poorest	2,354	22.8
Poorer	1,848	17.9
Middle	2,113	20.5
Richer	2,087	20.2
Richest	1,924	18.6
Total	10,326	100
Education of mother		
No education	4,273	41.4
Primary education	5,548	53.7
Secondary education	491	4.8
Higher education	14	0.1
Total	10,326	100
Education of father		
No education	2,070	20.0
Primary education	6,036	58.5
Secondary education	1,242	12.1
Higher education	49	0.5
Missing	922	8.9
Total	10,326	100
Occupation of mother		
Professional, technical, managerial, clerical	131	1.3
Sales, service, skilled manual	1,120	10.8
Agriculture, household chore, unskilled manual	6,867	66.5
Not working	2,206	21.4
Missing	2	0.3
Total	10,326	100
Occupation of father		
Professional, technical, managerial, clerical	578	6
Sales, service, skilled manual	4,002	39
Agriculture, household chore, unskilled manual	5,059	49
Not working	110	1
Missing	587	10.4
Total	10,326	100
Type of floor-material		
Natural (clay)	7,251	70.2
Rudimentary (wood and adobe)	190	1.8
Finished	2,629	25.5
Missing	256	2.5
Total	10,326	100
Type of toilet facility		
Flush toilet	183	1.7
Traditional toilet	5,615	54
No toilet facility	4,523	43.8
Missing	5	0.05
Total	10,326	100
Source of drinking-water		
Piped water	1301	12.6
Public tap	953	9.2
Well	4,048	39.2
Others	4,024	39
Total	10,326	100
Religious affiliation of mothers		
Catholic	2,620	25.4
Muslim	1,532	14.8
Zionist	1,200	11.6
Protestant/Evangelic	3,136	30.4
Animist/others	1,838	17.8
Total	10,326	100

The possible explanations for the observed differences in the present study might be related to the following:

First, Mozambique has geographically been divided into three main regions (North, Central, and South) but while this division was mostly derived from geographical and administrative factors, it had important implications on the economic structure of the country, with the South producing a much higher share of value-added activities than the Central and North (4). Currently, the inequality gap in development between the three regions have somewhat increased, especially with the emerging of mega-projects, such as the Aluminium Smelter Company and the Panda Gas in the South of the country in recent years (1). This unequal social development has also been accompanied with unequal distribution of the basic infrastructure, such as schools, hospitals, and water and sanitation systems (1), which, in turn, may play a role in the improvement of child welfare. Currently, half of the country's population does not have access to drinking-water and lacks adequate sanitation as well. A 2006 study found that the South provinces (Maputo, Gaza, and Inhambane) and Maputo city had the best access to the basic infrastructure and services while the Nampula province in the North and Zambezia and Sofala in the Central region had the worst access (4). In Mozambique, there is an uneven distribution of the basic infrastructure, social and economic factors, and healthcare (22). The distribution of some of these factors is shown in Table 2.

In the neighbouring country Zimbabwe, Root argued that the regional differences observed in child mortality could have been related to variations in the provision of healthcare and cultural factors (18).

Second, the differences in under-five mortality by province (region) of mother's residence might be related to lack of satisfaction in the basic needs of the poor population, although since 2005, reports of the Government on poverty reduction have indicated that the levels of absolute poverty have declined (1,2). For instance, Silva stated that the South's proximity to the Republic of South Africa, which provides a greater integration into the cash economy, may increase the ability of southern households (Maputo province and Maputo city) to accumulate more capital compared to the northern households, thereby contributing to inequality-increasing effects of trade (19).

The third potential cause for the observed differences in the mortality rates by province are the differences in cause-specific mortality. Although the 1997 and 2003 Demographic and Health Surveys did not collect data on causes of death, a recent study by the United Nations Children's Fund (UNICEF) found great differences in the distribution of the main leading causes of death (diarrhoea, HIV/AIDS, malaria, and acute lower respiratory tract infections—ALRI) among under-five children by province (3). The study also found that the Northern province of Cabo Delgado had the highest mortality rate of diarrhoeal diseases (192 per 1,000 people), followed by Zambezia province in the Central region with 169 per 1,000 people (3). Furthermore, the malaria mortality rates were high in Zambezia (569 per 1,000 people) and Cabo Delgado (539 per 1,000 people), and for HIV/AIDS, the highest under-five mortality was found in Gaza province in the South (268 per 1,000 people), followed by Zambezia (237 per 1,000 people) in the Central region. The mortality rates for ALRI were the highest in the Central provinces of Zambezia (282 per 1,000 people), Tete (238 per 1,000 people), and Cabo Delgado (225 per 1,000 people) (3). A study by Ghosh found pronounced differences in the prevalence of infectious diseases among under-five children in India, and he indicated the development of basic amenities, infrastructure, healthcare, and other macro-economic indicators as possible causes (13).

**Table. 4.** Risk ratios, with 95% confidence intervals for likelihood of under-five mortality in relation to province of mother's residence, 2003 Mozambique Demographic and Health Survey

Variable	Model I	Model II	Model III
	RR (95% CI)	RR (95% CI)	RR (95% CI)
	Unadjusted	Adjusted for demographic variables	Adjusted for socioeconomic/household environmental variables
Province of mother's residence (region)		
North region		
Niassa	2.11 (1.47-3.025)	2.09 (1.47-3.021)	1.94 (1.22-3.09)
Cabo Delgado	2.89 (2.03-4.09)	2.88 (2.02-4.08)	2.80 (1.77-4.43)
Nampula	2.35 (1.67-3.31)	2.25 (1.67-3.31)	2.15 (1.38-3.31)
Central region		
Zambezia	1.51 (1.04-2.20)	1.51 (1.04-2.20)	1.38 (1.04-2.19)
Tete	2.43 (1.73-3.42)	2.43 (1.73-3.42)	2.31 (1.73-3.42)
Manica	1.81 (1.26-2.58)	1.81 (1.26-2.58)	1.68 (1.09-2.56)
Sofala	2.35 (1.67-3.32)	2.35 (1.67-3.32)	2.12 (1.39-3.23)
South region		
Inhambane	1.62 (1.12-2.36)	1.62 (1.12-2.36)	1.61 (1.06-2.34)
Gaza	1.79 (1.25-2.57)	1.79 (1.25-2.57)	1.72 (1.12-2.54)
Maputo province	1.23 (0.82-1.85)	1.22 (0.82-1.85)	1.11 (0.70-1.77)
Maputo city	1	1	1
Demographic variable		
Mother's age (years) at child's birth		
24-28		1	1
18 years and less		1.41 (1.01-1.90)	1.40 (1.01-1.89)
19-23		1.09 (0.82-1.82)	1.09 (0.83-1.80)
29-33		0.86 (0.92-1.28)	0.83 (0.91-1.27)
34 and above		0.85 (0.69-1.06)	0.82 (0.64-1.04)
Birth order preceding birth interval		
Order 2-4 and medium interval (24-47 months)		1	1
First birth		1.96 (1.52-2.08)	1.90 (1.55-2.33)
Order 2-4 and shorter interval (<24 months)		1.07 (0.36-1.02)	1.06 (0.34-1.00)
Order 2-4 and long interval (48+ months)		0.55 (0.23-1.50)	0.53 (0.22-1.49)
Order 5+ and shorter interval (<24 months)		2.93 (1.60-2.40)	2.90 (1.55-2.39)
Order 5+ and medium interval (24-47 months)		1.70 (0.82-1.34)	1.69 (0.81-1.33)
Order 5+ and long interval (48+ months)		3.05 (2.08-4.13)	2.94 (1.98-4.07)
Sex of child		
Male		1	1
Female		0.50 (0.20-1.20)	0.40 (0.18-1.18)
Socioeconomic variable		
Place of mother's residence		
Urban			1
Rural			0.89 (0.53-1.09)
Wealth index		
Poorest			1.25 (0.86-1.81)
Poorer			1.27 (0.86-1.44)
Middle			1.26 (0.89-1.28)
Richer			1.28 (0.80-1.20)
Richest			1
Education of mother		
No education			0.40 (0.20-1.20)
Primary education			0.39 (0.15-1.11)
Secondary education			0.39 (0.12-1.14)
Higher education			1
Education of father		
No education			1.79 (0.97-1.85)
Primary education			1.21 (0.99-1.93)
Secondary education			1.17 (0.21-2.00)
Higher education			1
Occupation of mother		
Professional, technical, managerial			1
Clerical, sales, service, skilled manual			1.35 (0.44-1.60)
Agriculture, household chore, unskilled manual			1.23 (0.42-1.55)
Not working			2.65 (1.12-3.70)
Occupation of father		
Professional, technical, managerial			1
Clerical, sales, services, skilled manual			1.22 (0.97-1.85
Agriculture, household chore, unskilled manual			1.25 (0.99-1.94)
Not working			1.80 (0.80-1.20)
Type of floor-material		
Natural (clay)			0.98 (0.63-1.54)
Rudimentary (wood and adobe)			1.61 (0.97-1.68)
Finished			1
Source of drinking-water		
Piped water			1
Public tap			0.98 (0.56-1.45)
Well			1,26 (1.15-2.25)
Other			1.18 (0.58-1.90)
Religious affiliation of mother		
Catholic			1
Islam			1.01 (0.86-1.81
Zionist			1.11 (0.86-1.44)
Protestant/Evangelic			1.07 (0.89-1.27)
Animist/others			0.98 (0.23-1.20)

CI=Confidence interval;

1=Reference variable

Finally, we argue that the macro-economic factors, such as trade (national and international) and income distribution between and within the different provinces and the population density may, to some degree, influence their economic growth, which may indirectly affect the well-being of children. Although the impact of macro-economic factors on under-five mortality in Mozambique is beyond the scope of this study, the question regarding the economic growth and income distribution has been of great importance for low-income countries, including Mozambique (8). It is postulated that, in low-income countries, the growth is generally accompanied with a changing structure of economic activity: a transition from agriculture to industry and services (8). Thus, as real incomes rise during a period of economic growth, there is a danger that the gap between the rich and the poor can grow—something which can happen even if the income of the poor increases in real terms. Mozambique has experienced a considerable economic growth since the introduction of the market economy, especially through the implementation of the 'IMF Adjustment Program’ during the early 1990s, which somewhat increased geographical economic inequality (8,15). Furthermore, there is evidence that the rapid and unequal economic growth can, in a short term, be detrimental to the health of more vulnerable groups, including children (4,15).

In the multivariate analyses, controlling for the demographic, socioeconomic and household environment variables, the significance of the relationship between the province of mother's residence and under-five mortality only slightly reduced. It is possible that other variables (income distribution and trade, population density, distribution of basic infrastructure, including healthcare services, and climatic and ecologic factors) not included in this study could help explain the differences in mortality observed across the different provinces. Similar results were found by Uwazurike *et al*. for four sub-Saharan African countries where they reported unexplained spatial effects after controlling for an array of covariates (19). Interpreting the results, the author argued that climate and associated diseases and other macro-economic variables (population density and food insecurity associated with drought and flooding) were the likely reasons for unexplained patterns (19).

Mozambique has a tropical to subtropical climate, which is moderated by the influence of mountainous topography in the northwest of the country (23,24). There are seasonal variations in temperature with June, July, and August as the coolest months and December, January, and February the warmest. Generally, temperatures are higher close to the coast and in the southern lowland regions compared to the inland regions of higher elevation (23,24). The average temperatures in these lowland parts of the country are around 25-27 °C in the summer and 20-25 °C in the winter. The inland and northern regions of higher altitude experience average temperatures of 20-25 °C in the summer and 15-20 °C in the winter (23,24). Due to the country's location and climatic variations, it is prone to natural disasters, such as drought and flooding.

In a study of the impact of climate change on human health, Bambaige *et al*. noted that, although malaria (the main killer of under-five children in Mozambique) was prevalent in all provinces of the country, the provinces of Zambezia, Nampula, and Gaza reported the highest proportion of cases during 1998-2005 (24). On the contrary, Niassa reported the lowest proportion of cases during the same period. The increase was attributed to flooding which could have contributed to the growth of mosquitoes and the poor coverage of healthcare facilities (24). The study also found variations in cholera cases and diarrhoea (24). Climate variation and changes across the 11 provinces of Mozambique also affect food security (agriculture and livestock), which is essential for the well-being of the population, especially of mothers and children (23,24).

It is argued that the ecologic factors which include climate, soil, rainfall, temperature, altitude, and seasonality, can strongly influence child survival by affecting the quantity and disponibility of food-crops produced (23,24). Furthermore, it can affect the quality of water, various vector-borne diseases, the availability of income generated by work, and access to and use of medical facilities (23,25,26).

### Limitations

This study had some limitations. First, data from retrospective studies such as the 2003 DHS in general and birth-history data in particular are often subject to bias arising from faulty recall by respondents, which mostly is due to displacement of birth dates, misreporting of age at death, or simply omission of deaths, especially for infants who died early in life in the distant past (27,28). In addition, survival bias can occur since birth-history data are limited to the experiences of children born to surviving mothers (27). However, despite the above-mentioned caveats, several reports have found that the quality of DHS data is generally good to directly estimate the infant and child mortality rates and trends over time (27,28).

Second, it was not possible to include cause-specific mortality analyses by province, the availability of basic services and infrastructure (such as number of health centres, social services, schools, etc.), macro-economic factors (income distribution and trade by province), population density and climate and ecologic factors which are also important for the health and well-being of children.

Third, misclassification of province of mother's residence may have occurred. However, we believe that it is unlikely to have occurred to the extent of influencing the observed results since we used the *de jure* place of residence (where the women legally resided) instead of the *de facto* place of residence (where the women were interviewed regardless of their legal residence). Misclassification of other explanatory variables, such as occupation, may have also occurred. In Mozambique, many people who are considered unemployed and especially women are active in the informal economy where they sell various goods.

Finally, the study did not include data on healthcare-use in the analysis (which the survey collected only for under-three children) as its aim was to investigate mortality of under-five children (which is still very high both nationally and across provinces).

### Conclusions

Under-five mortality was associated with the province (region) of mother's residence. Children born to mothers living in the provinces situated in the North and Central regions had higher mortality risks than those born to mothers living in the South, especially Maputo province and Maputo city. However, controlling for the demographic, socioeconomic and environmental variables, the significance found between the place of mother's residence and under-five mortality reduced slightly, suggesting that other variables (income distribution and trade, population density, distribution of basic infrastructure, including healthcare services, and climatic and ecologic factors) not included in this study might also contribute to the observed differentials.

This study supports the thought that interventions aimed at reducing under-five mortality should be tailored to take into account the subnational/regional variation in economic development. However, research is warranted to further investigate the potential determinants behind the observed differences in under-five mortality.

## ACKNOWLEDGEMENTS

The study was supported by grants from University of Gävle and Karolinska Institute. The sponsors had no involvement in the study design, in the collection and analysis of data, in writing the manuscript, and in the decision to submit the paper for publication.
